# Joint study of genetic regulators for expression traits related to breast cancer

**DOI:** 10.1186/1753-6561-1-s1-s10

**Published:** 2007-12-18

**Authors:** Tian Zheng, Shuang Wang, Lei Cong, Yuejing Ding, Iuliana Ionita-Laza, Shaw-Hwa Lo

**Affiliations:** 1 Department of Statistics, Columbia University, New York, New York 10027, USA; 2 Department of Biostatistics, Mailman School of Public Health, Columbia University, New York, New York 10032, USA; 3 Department of Biostatistics, Harvard School of Public Health, Boston, Massachusetts 02115, USA

## Abstract

**Background:**

The mRNA expression levels of genes have been shown to have discriminating power for the classification of breast cancer. Studying the heritability of gene expression levels on breast cancer related transcripts can lead to the identification of shared common regulators and inter-regulation patterns, which would be important for dissecting the etiology of breast cancer.

**Results:**

We applied multilocus association genome-wide scans to 18 breast cancer related transcripts and combined the results with traditional linkage scans. Regulatory hotspots for these transcripts were identified and some inter-regulation patterns were observed. We also derived evidence on interacting genetic regulatory loci shared by a number of these transcripts.

**Conclusion:**

In this paper, by restricting to a set of related genes, we were able to employ a more detailed multilocus approach that evaluates both marginal and interaction association signals at each single-nucleotide polymorphism. Interesting inter-regulation patterns and significant overlaps of genetic regulators between transcripts were observed. Interaction association results returned more expression quantitative trait locus hotspots that are significant.

## Background

Breast cancer (MIM 114480) is a common and genetically heterogeneous human disorder [1]. Many studies have shown that gene expressions possess discriminating power for the diagnosis of breast cancer (e.g., van't Veer et al. [2]). Therefore, studying the genetic regulators of breast cancer related gene expression transcripts would shed light on the genetic mechanism of this disorder. Currently, the search for genetic regulators of expression traits in humans has been underway through linkage or association scans [3-5]. Morley et al. [4] measured the expression levels of 8500 transcripts using the Affymetrix Human Focus Arrays on 194 individuals in 14 Centre d'Etude du Polymorphisme Humain (CEPH) families. 3554 of these transcripts were identified to have greater between-subject variation than within-subject variation and were then used for linkage analysis. For the linkage analysis, genotypes of these CEPH individuals on 2882 single-nucleotide polymorphisms (SNPs) across the genome were obtained from The SNP Consortium .

In this paper, we study transcripts related to breast cancer susceptibility genes. Among the 3554 selected transcripts in [4], we identified 18 transcripts that are related to seven genes listed in the overview of breast cancer from OMIM (the Online Mendelian Inheritance in Man). Treating the expression levels of these genes as hereditable traits, we used multilocus association genome scans, combined with linkage scans, to study their regulation patterns and identify joint features and inter-relations. Gene expressions are likely to be complex traits that are regulated by multiple genetic factors. Therefore, we used an extension of our previous multilocus methods [6-8] to extract more association information.

## Methods

### Data processing

**Figure 1:**
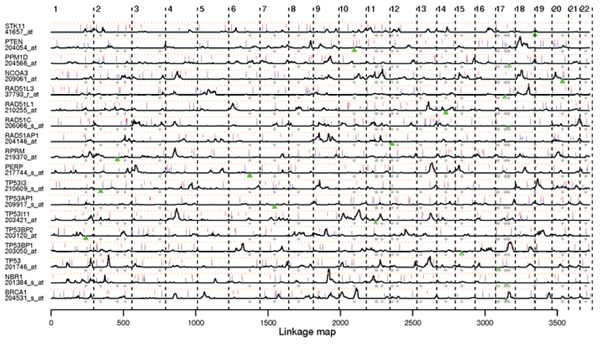
**Association and linkage scans for 18 breast cancer related transcripts**. Black curves are LOD scores from the linkage scans with the height of each row standardized by LOD = 5 and red dotted reference lines indicating LOD = 3. Top 30 SNPs with the strongest overall (blue ticks) and interaction (red ticks) association signal for a given expression trait are marked. A green triangle points out the genome alignment locus of the given expression sequence of that row, while gray dots are alignment loci of other breast cancer related expression sequences (including those were not studied in this paper).

For association and linkage scans, we studied the 2819 autosomal SNPs and the 18 transcripts listed in Figure 1. 86% of the SNPs have less than 10% missing genotypes and only 1.3% SNPs have more than 20% missing genotypes. Missing genotypes were imputed using fastPHASE [9]. For SNPs with weak linkage disequilibrium (LD) between them, the program is more likely to impute the most common genotype, which may affect the efficiency of our approach.

### Association scans

Extended from previously studied multilocus association scores on a dichotomous phenotype [6-8], a qGTD (quantitative genotype-trait distortion) statistic was proposed for quantitative traits of unrelated individuals [10]. qGTD is defined on the ranks of the trait values of *n *individuals, i.e., {*R*_1_,...,*R*_*n*_}. Given a set of *k *SNPs, there are 3^*k *^possible multilocus genotypes. The association content of this set of SNPs with the quantitative phenotype is then measured by

$$\text{qGTD}=\frac{12}{n^{2}(n-1)}\sum_{i=1}^{3^{k}}{\left(S_{i}-n_{i}\frac{\left(n+1\right)}{2}\right)}^{2},$$

where *S*_*i *_is the trait's rank sum on the *n*_*i *_individuals with genotype *i*, and *n*_*i*_(*n *+ 1)/2 is the expected value of *S*_*i *_under the null hypothesis that these SNPs are not associated with the phenotype.

qGTD captures the differences between the observed rank sums and those under the null hypothesis. The magnitude of qGTD scores reflects the level of association with the phenotype: the greater the value, the stronger the association [10]. Unassociated SNPs add dimensions to the multilocus genotypes and lower the value of qGTD. Therefore, a greedy screening algorithm is used to screen out SNPs that do not contribute to increase the value of qGTD and retain a *cluster *of SNPs that contribute important information to the score. As discussed in [6-8], such a screening is not informative for a large number of SNPs simultaneously due to sparseness in high dimensions. A random subspace strategy is then employed, where the greedy algorithm is repeated on a large number of random SNP subsets. SNPs are then ranked by the numbers of times (*return frequencies*) that they are retained by the screening algorithm, which measure the overall importance of individual SNPs.

To evaluate the importance of the SNPs in *gene *× *gene interactions*, we further filtered the retained SNP clusters from the qGTD screening by their qGTD scores and only selected the top 1000 distinctive clusters with the highest qGTD values. Using these 1000 clusters, we computed the *qGTD return frequencies *for each SNP. As discussed previously, higher value of qGTD indicates stronger joint effects from the SNPs on the quantitative phenotype. SNPs that present more frequently in clusters with higher qGTD values play a more critical role in gene × gene interactions that decide the variation of the phenotype.

In this paper, we apply the above association scan (repeated on 5 million random subsets) using the 56 unrelated grandparents in the 14 CEPH families. For each selected expression trait, we selected the top 30 overall important SNPs with the highest return frequencies, and the top 30 important interaction SNPs with the highest qGTD return frequencies, which give us a comparable number of identified loci as that by the linkage scans.

### Linkage scans

Linkage analysis was done on all 194 members of 14 CEPH families using the pedigree analysis package MERLIN [11]. The command pedwipe was first used to remove unlikely genotypes in the pedigree data. Regression-based linkage analysis for quantitative traits proposed by Sham et al. [12] was applied to all 18 expression traits with estimated mean, variance and heritability. The original data only contain physical map. In our analysis, we used linkage map provided by Sung et al. [13].

### Clustering of transcripts based on identified regulatory loci

To summarize the inter-regulatory-relation between the transcripts shown in the association scans, hierarchical clustering with average link [14] was conducted based on overall return frequencies, qGTD return frequencies, and common pairs of interacting loci. The dissimilarity measure for return frequencies (overall or qGTD) was 1 - correlation coefficient between two transcripts. For each transcript, we recorded the jointly returned SNPs in the 1000 qGTD-filtered clusters (see the section on *association scans*) and counted the number of times that SNPs that belong to a pair of loci (with loci defined as 5 cM bins on the genome) were returned in one cluster. The dissimilarity based on these interacting loci pairs is

$$D_{ij}=-\frac{m_{ij}-m_{i}m_{j}/m}{\sqrt{m_{i}m_{j}/m}},$$

where *m*_*ij *_is the number of shared interacting loci pairs between transcripts *i *and *j*, *m*_*i *_and *m*_*j *_are the total numbers of interacting loci pairs for *i *and *j*, respectively, and *m *is the total number of loci pairs on the genome. We also clustered the transcripts based on their gene expression values with dissimilarity being one minus the correlation.

## Results and discussion

### Combined association and linkage scans

Genome scan results for transcripts are arranged in the rows of Figure 1. Several interesting patterns are observed. First, association signals frequently cluster with linkage signals. Actually, similar to Roeder et al. [15], we may use linkage signals to control for false LD signals. However, since we only used single-locus analysis in the linkage scans, some loci may fail to have linkage signals if they are in interactions when deciding the traits' variation and thus have lower marginal signals. Second, both linkage and association signals show overlaps between transcripts that cannot be explained by chance, which is discussed in the next section. The performance of the association scan would also be greatly improved if denser SNP data were available.

### Transcription hotspots

**Figure 2:**
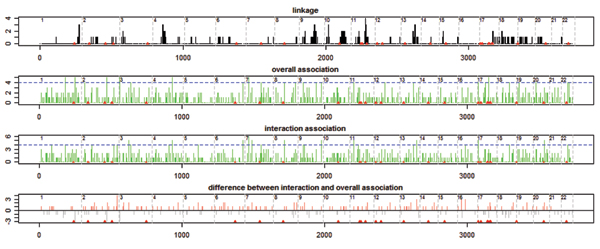
**Transcription hotspots identified by linkage and association scans**. Linkage, the numbers of times that a SNP has LOD > 1.44 (nominal *p*-value = 0.01) for a transcript were counted and plotted as black vertical lines. Association, the numbers of times that a SNP is one of the top 30 association SNPs for a transcript were counted. The SNP-by-SNP transcription hotspots pattern is noisy. To have a clear pattern, these counts were aggregated into bins of ≤5 cM by chromosomes as in Morley et al. [4]. Bins with ≥5 genetic regulators identified (*p *= 4 × 10^-3^) were identified as eQTL hotspots (blue dotted lines are the selection thresholds).

Figure 2 displays aggregated linkage signals and association signals for the 18 expression traits. Such overlapping genetic regulators patterns are sometimes referred as *hotspots *in the literature. The linkage signal is fairly clean as shown in Figure 2. Therefore, no aggregation by bins (as in Morley et al. [4]) was done. eQTL hotspots were identified as clustered black lines. For association, counts of identified SNPs were aggregated into bins of ≤5 cM by chromosomes and bins with more than five top SNPs were identified as eQTL hotspots (*p*-value = 4 × 10^-3^, evaluated using the Poisson model outlined in Morley et al. [4]). In Figure 2, loci of breast cancer susceptibility genes are marked with red triangles. The identified hotspots overlap with these genes: linkage at 2q, 11q, and 17q; overall association at 1q, 2q, and 17q; interaction association at 8, 17p, and 20q. Linkage and association have two identified genetic regulatory loci in common, the locus of BARD1 (MIM 601593) on 2q34-35 and the locus of BRCA1 (MIM 113705) on 17q21. Both loci harbor important breast cancer genes. The overall association scans incorporate both marginal and interaction signals and thus correlate better with the marginal linkage scans. Figure 2 also displays the difference between the interaction association signals and the overall linkage signals, which demonstrates that different regulatory loci have different extent of interaction activities.

Transcriptional hotspots may be observed even when the genotypic data are not linked to or associated with the phenotypes, due to reasons such as highly correlated phenotypes [17,18]. We calculated the correlation coefficients between the 18 transcripts studied and discovered that the highest value was 0.65. According to simulations, it is less likely to have false hotspots for traits with correlations at this level [18]. Therefore, what we have observed in this paper is more likely due to true regulatory activities.

### *cis*-Acting versus *trans*-acting

From Figure 1, not much evidence on cis-acting regulators has been identified except for some association signals and for the locus of BRCA1, which may require further investigations. Most interestingly, TP53BP1, a p53 binding protein, is shown to have a genetic regulator near the locus of BRCA1, a breast cancer susceptibility gene. It had been previously discovered by Rauch et al. [16] that 53BP1 binds to a promoter region of BRCA1 (MIM 113705).

### Clustering of transcripts

**Figure 3:**
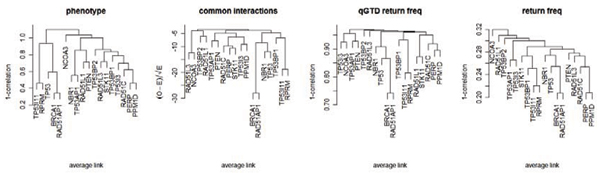
**Hierarchical clustering of transcripts**. Clustering is based on the phenotype (expression values), common interacting loci pairs, qGTD return frequencies, and overall return frequencies.

In Figure 3, we clustered transcripts using four sets of information (the phenotype, the number of shared interacting regulatory pairs, the qGTD return frequencies, and the overall return frequencies). BRCA1 and RAD51AP1 are found to share much more interacting regulatory loci than other transcript pairs. Also notably, the grouping based on interacting regulatory activities is different from that based on overall regulatory activities. Consistent clustering similarity between phenotype and association results was observed only on the strongest correlated pairs: BRCA1 and RAD51AP1, TP53I11 and RPPM. Such similarity is much weaker for the interaction association signals (shared interacting loci pairs and qGTD return frequencies).

## Conclusion

In this paper, we carried out a detailed joint study on 18 breast cancer related transcripts, using both linkage and association scans. Interesting inter-regulation patterns and significant overlaps of genetic regulators between transcripts were observed. Quantitative backward genotype-trait association (qBGTA), as a nonparametric multilocus association approach, studies eQTL without assuming a trait model while considering interactions [10]. Using qBGTA, we evaluated both marginal and interaction association signals at each SNPs locus and results on interaction association returned more significant eQTL hotspots (Figure 2).

## Competing interests

The author(s) declare that they have no competing interests.
